# Using written work to investigate stages in sixth-grade students’ construction and coordination of units

**DOI:** 10.1186/s40594-017-0085-0

**Published:** 2017-10-24

**Authors:** Catherine Ulrich, Jesse L. M. Wilkins

**Affiliations:** 1 School of Education, Virginia Tech, War Memorial Hall, RM 306, 370 Drillfield Drive, Blacksburg, VA 24061 USA; 2 School of Education, Virginia Tech, War Memorial Hall, RM 300C, 370 Drillfield Drive, Blacksburg, VA 24061 USA

**Keywords:** Assessment, Middle school student learning, Number sequences, Unit coordination

## Abstract

**Background:**

Students’ ability to construct and coordinate units has been found to have far-reaching implications for their ability to develop sophisticated understandings of key middle-grade mathematical topics such as fractions, ratios, proportions, and algebra, topics that form the base of understanding for most STEM-related fields. Most of the related research on unit coordination relies on time-intensive clinical interviews and teaching experiments. In this study, we investigate the work of 93 sixth-grade students on a written assessment containing whole number and fraction contexts using both continuous and discrete quantities, and how this work can be used to assess stages in students’ construction and coordination of units. Our investigation is guided by the following general research questions: (1) What forms of written work evidence the construction of and operation on composite units (units made up of other units)? (2) How does the categorization of students based on responses from a written assessment compare to written performance on a set of tasks conveying a continuous whole number multiplicative context?

**Results:**

We documented the different ways students represented composite units in their written work. In particular, student written work on tasks that included figurative unit items provided the greatest variety of evidence regarding students’ construction of and operation on composite units. However, written evidence from partitioning tasks did not seem as promising for distinguishing student stages. Students’ performance on decontextualized bar tasks involving continuous quantities was found to be consistent with students’ level of unit coordination based on written work providing evidence for the validity of stage categorizations.

**Conclusions:**

Our findings shed light on the affordances and constraints associated with particular stages in unit construction and coordination that a student brings to bear on tasks provided in a formal, written assessment. These findings provide promising evidence for scaling up the assessment of students construction and coordination of units through the use of written assessments instead of time-intensive clinical interviews.

Neo-Piagetian student learning researchers have long relied on time-intensive clinical interviews and teaching experiments in order to classify a student’s ways of operating. Recently, several teams of researchers have developed assessments that are meant to scale up the ability to make such classifications: Two examples include Clements et al. (2008) with a content focus on number, geometry, and classification with very young children and Hodkowski et al. (2016) with a focus on upper elementary multiplicative thinking. Of course, scaling up comes with an inherent trade-off in terms of the accuracy of classifications with regard to any one child. Nonetheless, the potential benefits from allowing a wider group of practitioners to make classifications or for allowing research with a larger group of subjects outweigh this drawback because the findings from studies with larger numbers of participants are generally seen as more generalizable and could potentially provide the evidence needed to inform curriculum and instructional policy and recommendations on a larger scale. We are currently in the early stage of developing another such written assessment, which looks at a content domain similar to Tzur and colleagues but focusing on sixth-grade students. In particular, we look at students’ ability to construct and coordinate units^1^ (Steffe, 2010a; Ulrich, 2015, 2016a), which has been found to have far-reaching implications for their ability to develop sophisticated understandings of key middle-grade mathematical topics such as fractions (Hackenberg & Tillema, 2009; Norton & Wilkins, 2009, 2010, 2012, 2013; Wilkins & Norton, 2011), ratios and proportions (e.g., Lamon, 2007; cf. a theoretical discussion in Thompson & Saldanha, 2003), and algebra (e.g., Hackenberg & Lee, 2015; Lee & Hackenberg, 2014), topics that form the base of understanding for most STEM-related fields. In addition to the Tzur research group, other written assessments have been used in research that focus on unit construction and coordination. So far, they have focused on particular contexts for unit coordination such as fractions (Norton & Wilkins, 2009, 2010, 2013; Wilkins & Norton, 2011) or whole number length problems (Norton, Boyce, Phillips, Anwyll, Ulrich, & Wilkins, 2015). The current study serves to set up a base of knowledge from which to refine a written assessment that incorporates both of these contexts, along with additional whole number contexts.

While many large-scale assessments are constructed as multiple-choice responses or focus mainly on the correctness of numerical responses, we are seeking to leverage the additional information that student-written work can give us. This would allow us to code not only for correctness, but also for the solution type. The trade-off is that the coding takes extra time and training when using the assessment. In developing our assessment, one of our first priorities is gaining a better understanding of how student solutions on written assessments evidence the construction and coordination of units, particularly the construction and coordination of composite units. Many of our items (and other researchers’ items) were based on items used in interview settings in which the interviewer can both provide and take away supports, such as the ability of students to draw diagrams, write down intermediate steps, etc. In this study, we are looking at what evidence of composite unit constructions we were able to glean when students had the chance to write but no additional supports from the researcher. In particular, we examine the types of student inscriptions that evidence composite unit constructions, and we look at the measurement utility of a series of length/area-based tasks that were not previously used by the authors in other settings.

## Theoretical framework

In carrying out this study from a neo-Piagetian perspective, we assumed that student mathematical thinking develops in hierarchical stages, in each of which some basic ways of operating have been adapted to underlie a variety of mathematical schemes. In particular, we find the stages in student construction and coordination of units (Steffe, 2010a; Ulrich, 2015, 2016a) to be important for exploring the mathematics of middle-grade students. These stages were originally developed when working with students on situations involving discrete, whole number quantities: Steffe and colleagues (e.g., Steffe & Olive, 2010; Steffe & Cobb, 1988) proposed a hierarchy of stages called *number sequences*, which characterize how students work with units and their coordinations when operating on what we would call counting numbers. *Units* refer to interiorized counting acts, so they can be used to enumerate the size of sets of visible or invisible items and can themselves be counted. Steffe and Olive (2010) have also elaborated how the operations that characterize these number sequences are reorganized to form fraction schemes. Therefore, these number sequences can now be used to help predict both whole number and fractional operations that are within a student’s zone of potential construction (Norton & D’Ambrosio, 2008; Steffe, 1991). The types of number sequences, in order of increasing sophistication, are (1) initial number sequences (INS), (2) tacitly nested number sequences (TNS), (3) explicitly nested number sequences (ENS), and (4) generalized number sequences (GNS). In our written assessment, we are attempting to differentiate between students at the stage associated with tacitly nested number sequences (TNS students) and the stages before and after this stage. The following discussion of number sequences will provide reasons for this focus.

**Fig. 1:**
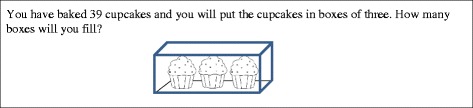
Cupcake Task A (For this task and all other tasks in this study involving cupcakes, the cupcake image is used courtesy of MyCuteGraphics.com © Laura Strickland)

We will briefly give an overview of the key constructions associated with the stages associated with these first three number sequences (see also Table 1): The construction of INS is associated with the first truly numerical stage in that students now see numerosity as an attribute that describes the size of a set. The construction of an INS means that students can make sense of a number word without first counting from 1 up to that number word. This can be seen, for example, when a student can count on from a given number by a given amount instead of needing to reestablish the meaning of the first number by counting from 1 (counting all) each time. In developing our written assessment, we would expect that all our participants would have constructed INS by sixth grade due to our previous experience with sixth-grade students and Steffe’s estimate that (almost) all students will have constructed an INS by third grade (Steffe & Ulrich, 2014). Students who are at the stage associated with INS (INS students) typically depend on visuospatial or rhythmic patterns to count non-initial number sequences. For example, when solving Cupcake Task A (Fig. 1), an INS student would have trouble keeping track of the number of times he or she counts 3 cupcakes when counting 39 cupcakes in his or her head. We would expect an INS student to draw out 39 figurative items, go back and circle subitized groups of 3 using visuospatial patterns, and then count the groups of 3.

**Table 1:** Summary of key stages investigated

Stage		Key characteristics	Possible indicators
INS		• Numerical thinking • Use of numerical composites	• Counting on • Reliance on figurative material to keep track of composite quantities
TNS	eTNS	Construction of composite units during problem-solving activity	• Constrained to equi-segmenting operations when partitioning • Need to construct composite units in new problem situation before they can be used
	aTNS	Anticipatory use of composite units	• Simultaneous partitioning operations • Ability to mentally work with composite units • Difficulty keeping track of multiple quantities in a problem situation
ENS		• Construction of iterable units of 1 • (Reversible) disembedding • Coordinating three levels of units in activity	• Equi-partitioning operations • Construction of fractional schemes • Ability to relate multiple levels of units mentally

Over time, a student would develop the ability to reflect on the numerical composites formed by experientially bounding counting-on activity. For example, after counting from 8 to 12 many times, the student would develop a tacit understanding of 8 as being nested in 12 and a tacit understanding of the numerosity of the counting acts from 8 to 12—“9, 10, 11, 12”—as representing how much larger 12 is than 8. This reconceptualization of counting-on activity results in a reorganization of initial number sequences into tacitly nested number sequences. It can take more than 2 years for students to reorganize one number sequence to construct the next (Steffe & Cobb, 1988).

The construction of a TNS is marked by constructing *composite units* of counting acts (like the composite unit of 4 that represented the subsequence from 8 to 12) that can be used in further operations (like the repeated use of “counting by 3” as a unit of measure in Cupcake Task A). In general, *composite units* are units composed of multiple smaller units such that the student can treat them as a single unit while remaining aware of the constituent units contained within it. Therefore, when solving Cupcake Task A, a student who has constructed a TNS can form the goal of figuring out how many times they count by 3, a composite unit, to get to 39. In general, use of a TNS would be indicated by skip counting or repeated addition to solve multiplication or division problems^2^ (Olive, 2001; Ulrich, 2015).

In Steffe and Cobb’s (1988) study with K-2 students in which the number sequences were first theorized, operating with a TNS was thought to be a transitional stage that students would move through quickly as various tacit quantitative relationships were made explicit upon repeated exposure and reflection. In Steffe and Olive’s (2010) work with grade 3–5 students, the stage associated with construction of a TNS clearly lasted many months but was still seen as transitional. In working with middle-grade students (Ulrich, 2012; Ulrich & Phillips, 2015), we have begun to suspect that some students remain at this stage for multiple years: We have found students who can utilize composite units in unexpected ways, implying a great familiarity with composite units, and yet have not yet moved to the next stage of unit construction and coordination (Ulrich, 2016b). One indication of a student’s familiarity with composite units is an ability to anticipate the results of constructing composite units before they construct them. For instance, this student would be able to form the goal of counting by 3s to 39 *before* constructing a composite unit of 3 to stand in for a box of cupcakes in Cupcake Task A. Despite this anticipatory use of composite units, the fact that they are constrained to the stage associated with the TNS is made clear through several serious constraints in their mathematical thinking (see Ulrich, 2016b). Because we are interested in what percentage of middle-grade students are constrained to these kinds of advanced operations with a TNS, we broke the stage associated with a TNS into two substages when designing the written assessment: (1) students who are still early in the stage (eTNS students) and (2) students who can do advanced operations within this stage (aTNS students).

One result of the fact that aTNS students can operate fluently with composite units is that they can use composite units as a template for partitioning a whole into small numbers of equal parts. INS and eTNS students would, in contrast, attempt to *equi-segment* (Steffe, 2010b) a fractional whole by making a guess as to the size of one segment and then using that segment as a template for measuring off further pieces. This generally results in slow and inaccurate partitioning operations. An aTNS student can mentally visualize a composite of continuous units, project that onto the unpartitioned whole, and then mentally adjust the sizes of the continuous units in the composite to get a much better initial estimation than an eTNS student would. This is called *simultaneous partitioning* (Hackenberg, Norton, & Wright, 2016; Steffe, 2010b) because of the simultaneous awareness of all the partitions before the student acts on the visible whole.

**Fig. 2:**
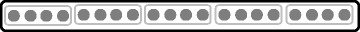
Twenty as a composite unit made up of 5 composite units of 4

**Fig. 3:**
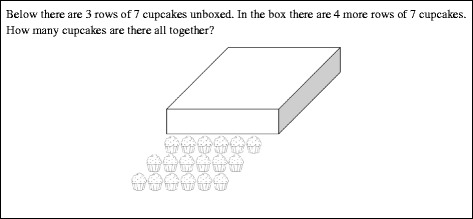
Cupcake Task B

Finally, students who have constructed an ENS are characterized by the development of a reversible *disembedding* operation and an *iterable unit* of 1 (Ulrich, 2016b; cf. Steffe, 1992, 2010a). The iterable unit allows students to reconceptualize their number words to a single multiplicative relationship instead of a sequence of counting acts. For example, 5 can be thought of as five times 1 instead of a sequence of five distinct counting acts. This ability to hold in mind 1 of the five units in 5 and then compare it to its containing unit of 5 is indicative of the more general act of *disembedding* that characterizes ENS operations. In general, students can utilize an ENS to simultaneously consider a composite unit and one of its component parts and compare them, even if that component part is itself a composite unit. For example, a student at this stage might note that a composite unit of 20 is made up of 5 composite units of 4 (see Fig. 2). While a TNS student may be able to determine that they count by four 5 times to get to 20, the ENS student can actually reconceptualize 20 as being made up of 5 copies of a composite unit of 4 in the course of solving a problem. This is a subtle distinction, but it allows ENS students to more frequently operate on the results of iterating composite units during problem-solving activity (Steffe, 1992; sometimes called constructing *three levels of units in activity*, see Hackenberg & Tillema, 2009). The efficiency in conceptualizing whole numbers as iterations of a single iterable unit is thought to allow many of these more advanced mathematical constructions students make after construction of an ENS. Therefore, students at this stage would not only be able to solve Cupcake Task A (see Fig. 1), but they would be able to then operate on their 13 groups of 3s to add or subtract boxes. We would also expect ENS students to be able to solve a problem such as Cupcake Task B (see Fig. 3) with very little, if any, written work.

**Fig. 4:**
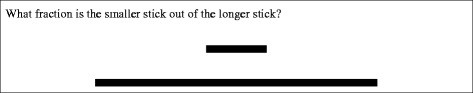
Example of a task used to elicit a student’s partitive unit fraction scheme

**Fig. 5:**
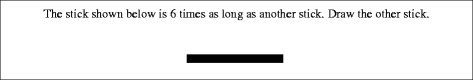
Example of a task used to elicit a student’s splitting operation

The ability to understand composite units as multiplicatively related to a unit of 1 is mirrored by the ability of ENS students to understand the results of their partitioning activity as unit fractions in a multiplicative relationship with the fractional whole: a student can use a unit fraction “as if it were an *iterable fractional unit* that was on par with his iterable unit of 1” (Steffe, 2010c, p.101). This type of partitioning is called *equi-partitioning* (Hackenberg, Norton, & Wright, 2016; Steffe, 2010b). In contrast, simultaneous partitioning results in partitions that are equal in size but are not seen as potentially resulting from iterations of each other by the student. Therefore, the simultaneous partitioner would think about the fractional whole as being made of *n* different but congruent partitions while the equi-partitioner would construe the fractional whole as *n* times as long as the partition and/or the partition as 1/*n* times as long as the fractional whole. The resulting way of viewing fraction notation is called a partitive unit fraction scheme (see Steffe, 2010b, 2010c 3; see Fig. 4 for an example of a task that students are not usually successful on until they have a PUFS, e.g., Norton, Wilkins, & Xu, in press). Once the ENS student is aware of both inherent multiplicative relationships before creating the partitions, we say that the student has constructed a *splitting operation* (Hackenberg, 2007; Steffe, 2010b; Wilkins & Norton, 2011). For example, the task in Fig. 5 would elicit a student’s splitting operation. In order to solve this task, a student would need to recognize that although the wording of the task implies iteration, the solution requires partitioning the whole. In contrast to equi-partitioning and the partitive unit fraction scheme, the student’s operations of partitioning and iterating occur simultaneously when using the splitting operation (Steffe, 2002; Wilkins & Norton, 2011).

We will not differentiate between construction of ENS and GNS in this study, so an *ENS student* will throughout imply the construction of at least an ENS but does not imply that the student is limited to that number sequence construction.

## Purpose of study

The purpose of the analysis reported in this article is to gain a better understanding of specific aspects of students’ written work on our assessment that could be useful as evidence of student constructions and coordinations of units. Our investigation is guided by the following general research questions: (1) What forms of written work evidence the construction of and operation on composite units? (2) How does the categorization of students based on responses from a written assessment compare to written performance on a set of tasks conveying a continuous whole number multiplicative context? Answering the first research question provides a better base for coding student solutions in future iterations of similar written assessments. The second research question tests the association between a category of items that have increasingly been used in written assessments of unit coordination and items that the researchers had previously utilized in clinical interviews and teaching experiments. All of this information will help with revisions of a written assessment that will allow a scaling up of the more time-intensive clinical interviews that are usually used to determine a child’s number sequence constructions.

## Methods

### Participants and overview

The analysis described here is based on data from the administration of the first version of a written assessment of students’ stages of unit construction and coordination. This written assessment was administered to 109 sixth-grade students from five sixth-grade classrooms in the same school all taught by the same teacher. One classroom was identified as Advanced (*n* = 26) and the other four classrooms were identified as Regular. The school is in a small town in the southeastern USA. Of these 109 students, 15 did not give written consent for their data to be used in analysis. One additional student provided written consent but did not complete the assessment due to schedule conflicts. The data from these 16 students were removed from the study resulting in a working sample of 93 students.

As part of our ongoing validation and improvement of our written assessment, the second author chose nine students from the 93 to participate in follow-up clinical interviews carried out by the second author. The first author then used the clinical interview data to classify the nine students and gain information on how students were thinking about various items. This data has been used to inform future iterations of the written assessment. These nine students were chosen based on written parental consent and distribution across the five classes. All research procedures were approved by our institution’s Institutional Review Board for research involving human subjects.

We classified students’ stages of unit construction and coordination based on their responses in the written assessment using a previously developed coding and scoring system. Our first research question was answered through a qualitative analysis of the written responses on the assessment, and our second research question was answered through a comparison of performance on various items in the written assessment.

In the following sections, we will describe our initial development of the written assessment and scoring system as well as our analysis methods for the specific research questions we are examining in this article.

### Development of written assessment

The development of our written assessment and associated coding guide and scoring rubric proceeded through several stages. We describe the stages of this process here. We include a detailed discussion of specific types of tasks in the next section.

#### Initial task development

Our goal in developing the assessment instrument was to provide a battery of tasks from a variety of contexts to assess students’ current stages of unit construction and coordination, associated with the number sequences described earlier (INS, eTNS, aTNS, and ENS/GNS). Based on previous research on number sequences (e.g., Steffe, 1992; Steffe & Olive, 2010; Ulrich, 2012), we designed or selected tasks for the written assessment that would invoke students’ actions associated with each of the number sequences. Many of the tasks allow for multiple types of written work, regardless of whether the final answer was correct or incorrect. For example, in Cupcake Task B (Fig. 3), students could write the answer with no work shown; they could draw out all the hidden cupcakes or they could just represent hidden rows. In addition, multiple tasks were included that could indicate or contraindicate various stage constructions. We wanted to limit the number of questions so that a sixth-grade student could be expected to comfortably complete all questions in a 50-min class period. In the end, we developed 25 tasks covering the following general types of questions: discrete versus continuous unit items, whole number versus fractional values, problems involving (to the observer) multiplicative situations, or problems involving only additive situations. The different types of tasks were distributed across the entire assessment. However, the assessment was written to be administered in two parts (11 tasks in the first part and 14 tasks in the second part), to prevent students from returning to some tasks intentionally placed earlier (i.e., in Part I) in the assessment to test for more advanced ways of thinking. For example, a task in Part I might require students to work with composites of composite units (e.g., see Fig. 9, to be discussed later), whereas a similar task might require less sophisticated reasoning with composite units (e.g., see Fig. 3). These two tasks were purposely placed in Part I and Part II, respectively, in order to control for potential learning during the test, in which case, students could potentially return and make changes to the earlier tasks if they were not administered separately.

#### Development of the coding guide

The research base was also used to inform the design of a preliminary coding guide that identifies types of solution strategies for each task that could be useful in making stage classifications later on. Next, we asked a mathematics educator who is an expert in the theory to review the tasks on our written assessment and the initial codes associated with each task to confirm that the tasks were measuring what we intended. Tasks and codes were revised after this consultation.

#### Development of the scoring rubric

Finally, formulas were created for each of the number sequences: ENS/GNS, aTNS, eTNS, or INS (or less advanced). These formulas use the task codes to determine whether or not construction of the particular number sequence in question should be attributed to a student. We refer to the set of classification formulas as the *scoring rubric* and to the result of the classification formulas as the *stage classification*.^4^ The formulas take into account both indications and contraindications, weighted by differing strength of inference—strong (weighted 1), moderate (weighted 0.6), or weak (weighted 0.3)—based on prior research (Steffe, 1992; Steffe & Olive, 2010; Ulrich, 2012). Indications were given a positive value and contraindications were given a negative value. The result of this strategy is that one strong indication will outweigh multiple weak contraindications. In the end, our intent was for the student’s most complex uses of composite units to determine the stage classification. However, even strong inferences are still just inferences. Therefore, we thought it was important that, as in a clinical interview, a pattern of strong contraindications be given importance as well.

As an example of how indications and contraindications can play out in the stage classification of a particular student, consider the work of Student 4. Student 4 exhibited three, related weak contraindications of having constructed an aTNS by drawing in all the items in three fairly easy tasks, including Cupcake Task A and another task that required simple subtraction. However, these contraindications were outweighed by one strong, one moderate, and one weak indication of having constructed an aTNS. The strong indication was the correct solution of two out of three splitting tasks (the splitting operation is not available until after the construction of equi-partitioning; aTNS students can solve splitting tasks through the strategic use of simultaneous partitioning, see Ulrich, 2016b for an example). The moderate indication was a correct solution to Cupcake Task B (involving operating on the results of a unit coordination) without showing any work to support the unit coordination needed to enumerate the hidden cupcakes. The weak indication was a characteristic conflation when trying to name a fraction based on an incomplete partition that shows an awareness of multiple levels of units but an inability to keep track of them. Therefore, the stage formula for aTNS indicates that Student 4 should be classified as having constructed an aTNS.

In contrast, Student 4 showed no indications of having constructed an ENS but did show three weak contraindications. One involved an incorrect solution when naming a proper fraction based on an incomplete partitioning. The other two involved incorrectly solving tasks that were meant to require operating on the results of a unit coordination. One of these tasks was Cupcake Task C (note that answering the wrong question and giving the total number of cupcakes was not coded as incorrect, so the error was more fundamental) and a problem involving ice cream sundaes with smaller numbers than Cupcake Task C but no picture. Therefore, the stage formula for ENS indicated that we should not attribute the construction of an ENS to Student 4. This means that the stage classification for Student 4 was aTNS.

As one can imagine, students will sometimes fail to use advanced schemes and operations at particular times or in particular contexts even though they have constructed and used these schemes and operations at other times or in other contexts. As a result, the written data from the assessment can only be interpreted as providing positive evidence or absence of evidence when classifying students’ number sequence. These classifications as determined by the scoring rubric should be understood as a lower bound. Therefore, our assessment could underestimate the sophistication of students’ constructions on any particular day.

#### Administration of the written assessment

The finalized written assessment was administered to students in May 2015. After data collection was complete, the written assessments were de-identified by removing student names and labeled with a number. This was done to mask the identification of the students during the analysis of the clinical interviews, described below.

#### Coding and scoring of written assessment

We randomly selected and coded 10 of the 93 written assessments (approximately 10%) using the coding guide. The first author then did a face-value check of the task responses to give a qualitative analysis of the stage classification for these 10 students. We then compared, discussed, and reconciled our task codes. Based on this discussion, we reworded the coding guide for coding practicality and consistency. We also used the scoring rubric to make stage classifications for these 10 students and compare those with the qualitative stage classifications of the first author. Based on this exercise, we revised some of the indicators or weights used in the scoring rubric to maintain consistency with the theory associated with number sequences (e.g., Steffe, 1992; Steffe & Olive, 2010; Ulrich, 2016a, b). For example, we had not anticipated that students would actually partition a bar into 21 pieces to successfully make a bar of length 14 based on a bar of length 21. Therefore, we had inappropriately made successful completion of this task a strong indication of ENS when, in fact, an aTNS student could solve by partitioning into units of 1. We adjusted the scoring formula for ENS to distinguish between different correct solutions to the task.

We then independently coded the remaining 83 students and calculated stage classifications for both sets of codes.

To assess the overall agreement (inter-rater reliability) for student classifications (i.e., ENS, aTNS, eTNS, or INS) we calculated a weighted kappa statistic, *K*, for the 83 students. The weighted kappa statistic for stage classification is 0.95 which represents “almost perfect” agreement (Landis & Koch, 1977, p. 165). This estimated kappa was not found to be due to chance (*p* < .05 for the significance test). This statistic provides evidence of strong reliability for rater classifications based on the written assessment.

We discussed and reconciled any disagreements to create one final classification for each student. Of the 93 students, 44 were classified as ENS; 39 were classified as aTNS; 6 as eTNS; and 4 as INS. Including all 109 students surveyed, 40% were classified as ENS; 36% as aTNS; 6% as eTNS; 4% as INS; and 15% did not participate.

#### Analysis of clinical interviews

Structured clinical interviews were conducted with nine students by the second author and analyzed by the first author (student identification was masked in order to maintain anonymity of student work during analysis and subsequent comparison with the written assessment). The interviews followed a structured protocol involving a smaller selection of tasks similar to those found on the written assessment. The goal of these interviews was to provide a baseline indication of the potential for the written assessment to be used for categorizing students into the stages. Based on the students’ responses and actions, the first author was able to make a strong inference as to the stage classification for six of the nine students. For the other three students, the first author gave a range of two classifications. We would later find that the scoring rubric gave the same classification or a classification within the designated range for eight of the nine students.

Before looking at the classifications from the written assessment, the research team further discussed the three inconclusive students to settle on a final classification. The assessment classifications matched the clinical interview classifications for five out of the six students with conclusive classifications and one out of the three students with inconclusive classifications, representing a moderately strong relationship between the two classifications. The one student who had a conclusive classification from the clinical interviews that did not match the classification based on the written assessment showed strong indications of ENS during the interview but was classified as aTNS based on the written assessment. We examined her written work and found one task answered incorrectly on the written assessment that would have given a decisive indication of an ENS. In the interview, she drew a correct answer for this same task and provided a correct explanation.

#### Discussion of reliability and validity

Through this step-by-step process, we gained confidence that our written assessment was measuring what we had intended. This process enabled us to provide evidence for the reliability of the stage classifications based on the written instrument. In addition, the alignment of our tasks with prior research and the review and consultation with an expert in the field provides evidence for the face validity and content validity of the written instrument and associated coding guide and scoring rubric. Finally, the comparison of the stage classifications from the clinical interviews with those based on the written assessment provides evidence for the predictive validity of the stage classifications based on the written assessment.

### Description of tasks

We will now provide a detailed description of the various types of tasks included in the assessment.

#### Tasks assessing fraction schemes and operations

The tasks borrowed or modified from previous written assessments of students’ fraction schemes and operations included four tasks that elicit a student’s partitive unit fraction scheme (e.g., Fig. 4; Steffe, 2010b) and four tasks that elicit a student’s splitting operation (e.g., Fig. 5; Hackenberg, 2007; Steffe, 2010b; Wilkins & Norton, 2011), both of which imply a construction of an ENS (Steffe, 2010b), and two tasks that elicit students’ partitioning operations (see Norton & Wilkins, 2012, 2013; Wilkins & Norton, 2011; for further discussion of these tasks). Note that we avoided the use of powers of 2 in tasks involving partitioning because that partitioning can be carried out by a series of halving actions, which is available to students before simultaneous or equi-partitioning operations are available. Furthermore, students will sometimes be able to use small numbers, such as 2, 3, and 4, in much more complex ways than the other numbers in their number sequence, so behavior when partitioning with 3 and 4 may be more advanced than other partitioning behavior. For that reason, we used a variety of number of partitions, including 3, 4, 5, 6, 7, and 9, with multiple problems using partitions of 5 and 6. The partitioning tasks were only coded based on whether there were an appropriate number of partitions and whether those partitions were approximately equal in size.

In addition to these tasks, we adapted one other fraction task from clinical interview protocols in which students have to interpret the results of partitioning a partition. In clinical interviews and teaching experiments, the use of appropriate fractional language has always indicated the construction of at least a partitive fraction scheme (Ulrich, 2016b; Steffe, 2010b).

#### Tasks involving discrete composite quantities

Two tasks involving discrete quantities presented an unknown number of marbles being added to a cup of marbles. These tasks were meant to help differentiate between INS and eTNS. The rest of the tasks involving discrete quantities required multiple applications of a composite unit. In Cupcake Task A (Fig. 1), the composite units were boxes. In Cupcake Tasks B (Fig. 3) and C (Fig. 9), the composite units were rows. In both situations, there were also boxes that represented a composite of composite units (3-level unit) that had to be operated on in some way. There were two other problems involving composite discrete units. One involved Legos made into Lego houses. The other involved scoops of ice cream in sundaes. In many of these problems, the ability of a student to do the problem mentally without writing down any intermediate steps or supporting work was taken to indicate a higher number stage construction. However, we recognize that some students might routinely show their work because of teacher expectations and not out of need for figurative material. Therefore, we do not count the use of figurative drawings (e.g., drawing out cupcakes) “against” students for higher stage attributions (i.e., give a negative score for this work). For example, if they do some of the more complex problems mentally, that would indicate ENS, but if they show work, it does not contraindicate ENS.

#### Task for partitioning a composite continuous quantity

There was one task meant to provide an opportunity for students to show whether they assimilated tasks with composite units or three-level units. In this task, one bar was said to be 14 units long and the student was asked to draw a bar that was 21 units long. If the student were to utilize 7-unit lengths to draw the bar, then they would show that they could think of 14 and 21 as made up of multiples of composite units before operating and utilize that knowledge to plan a solution strategy. That would, in fact, imply a stage higher than the ENS stage but certainly would be a decisive indication of ENS. Simply solving that problem would require assimilation with composite units, which implies at least an aTNS.

Note that in this and other problems, a student’s lack of fluency with multiplication would make the more sophisticated solution strategy unlikely, even though they might have constructed an ENS or GNS. Nonetheless, we find these items valuable in that it gives students who are relatively fluent in multiplication to give indications that would help us differentiate between aTNS and ENS. As such, the fact that a student does not use the most sophisticated strategy to solve this or another problem does not by itself count against the student in determining a given stage construction. However, it can give a decisive indication that will count strongly towards attributing the more sophisticated number sequence construction.

#### Bar tasks

**Fig. 6:**
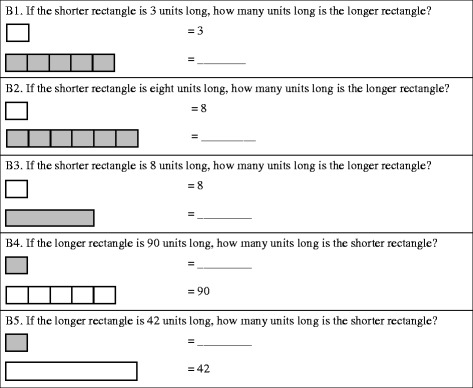
Bar tasks

There were six tasks on the assessment that show two bars such that the length (and area) of one bar is a whole number multiple of the other. Sometimes partitions are used to show the iterations of the smaller bar in the larger bar, and sometimes they are not. In all tasks, the length of one bar is given and we ask for the length of the other. In all five tasks analyzed in this paper, the smaller bar’s length is itself a composite unit. We refer to these tasks as the *bar tasks*. We used five of these tasks in our analyses (see Fig. 6). Based on the kappa (*K*) statistic, overall rater agreement (inter-rater reliability) was either perfect or almost perfect (Landis & Koch, 1977) for the five bar tasks: B1(*K* = .95); B2 (*K* = 1.00); B3 (*K* = 1.00); B4 (*K* = .98); B5 (*K* = .93), representing high reliability for rater agreement. These tasks are not dissimilar to those used by other researchers to study unit coordination (Kosko & Singh, in press; Norton, Boyce, Phillips, Anwyll, Ulrich, & Wilkins, 2015; Norton, Boyce, Ulrich, & Phillips, 2015), but they are different enough that we did not feel comfortable using them to categorize students according to the stages of unit construction and coordination. Therefore, we instead developed some hypotheses regarding their difficulty relative to each other and relative to the other tasks that we can now test.

### Data analysis methods for the current study

To answer the first research question, the first author noted tasks with a variety of student work that illustrate students’ use of composite units. All of the promising tasks involved discrete units or partitioning. She chose one task involving discrete units and one partitioning task to fully analyze. For these two tasks, question by question, she laid out all responses from students who had received an INS classification based on the assessment rubric, all responses from students who had received an eTNS classification based on the rubric, the first 10 responses from students categorized as aTNS, and the first 10 responses from students categorized as ENS. Throughout the remainder of the paper, we will use the term *probable* to refer to classifications based on the assessment rubric. She then identified all distinct types of written evidence for the use of composite units (or contraindications), selecting student responses that exemplified these types of written evidence. For each of the selected exemplars, the first author then went through each assessment in full to qualitatively determine whether the student responses seemed representative of what she would expect of a student with the assigned stage classification. When her classification did not match up with that of the assessment rubric, she randomly selected other responses at the given stage until she found one in which the classifications did match up. In that way, we feel fairly confident about the stage classifications of each student whose work is shown in the “Results and discussion” section. She additionally looked through all other student responses to these questions to find any additional types of written evidence. Finally, she looked through all 33 of the originally chosen assessments in full to see if there were other types of written evidence that were not included in selected student solutions to the original two tasks. There were, and so the same procedure was carried out with an additional task of each type (discrete unit and partitioning). In the end, all types of evidence were covered using both discrete unit tasks and the second partitioning task.

With regards to the second research question, we hypothesized the following hierarchy and correspondences between the bar tasks (Fig. 6) and stage classifications: (H1) The bar tasks will follow a hierarchy of increasing difficulty—B1, B2, B3, B4, B5; (H2) success rates on each task will be positively associated with stage classification; (H3) B1 would be possible for some students who had not yet constructed composite units (INS); (H4) B2 would be possible for students who had constructed composite units (eTNS); (H5) B3 and B4 would be possible for students who could assimilate using composite units (aTNS); and (H6) B5 would be possible for students who had constructed iterable units (ENS). Evidence for the hierarchy was based on percent correct for each task. Additionally, the magnitude and patterns of association between student performance on each of the five bar tasks and stage classification was assessed using the gamma statistic (*G*). *G* is appropriate for assessing the association between two ordinal measures (e.g., B1 versus stage classification; Siegel and Castellan, 1988). Hypotheses 3 through 6 were evaluated through an investigation of the associated contingency tables. Given evidence of an association between task and stage, step-like patterns in the tables were used to identify the classification for which tasks were accessible.

## Results and discussion

For the purposes of the present study, we were primarily interested in finding out what aspects of student-written work are most promising as evidence of constructing and operating on composite units and how performance on the written assessment compared to performance on a battery of decontextualized tasks involving length (i.e., the bars tasks).

### Evidence of construction of and operation on composite units

While we can always look at whether students got a problem wrong or right as an indication of certain ways of operating, we chose to feature student responses that would highlight the range of responses involving figurative representation of or inferred internalized use of composite units in solving the tasks. The focus of the discussion of the responses in this section are not meant to reflect classification strategies as student responses to a single item are not sufficient to classify students. Any stage constructions we attribute to students in the “Results and discussion” section are based on their overall performance on the assessment and a qualitative analysis of the entire assessment by the first author. The purpose of the responses we share here is to highlight aspects of student written work that indicated the construction of and operation on composite units. In particular, we will highlight two ways that this showed up in written responses: partitioning and operating on composites of composite units.

#### Partitioning

In our coding for the assessment, we were only focused on whether the student had formed the correct number of partitions and whether the partitions were approximately equal in size. However, within our 93 student responses, we noticed a variety of written evidence regarding the use of composite units while partitioning that we wish to share with the reader. Many of these pieces of evidence regard subtle distinctions in student work and inferences about the order of student work; therefore, we have yet to determine which of them may be feasible to bring into our codings for future assessments. It is more likely that these indications will be useful for smaller scale studies in which researchers can record the sequence of student-written work.

**Fig. 7:**
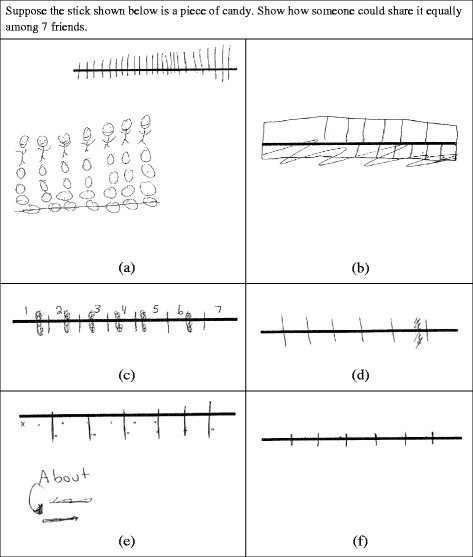
Six solutions to a partitioning task

Figure 7a shows a probable INS student who shows no evidence of using a composite unit to complete the task. This student segmented the bar into a bunch of fairly equally sized small pieces (i.e., 26 pieces). Once the bar had been segmented in this way, the student appears to draw a diagram that represents divvying out these pieces to seven stick figures. Note that the number of pieces created by segmenting the bar is not a multiple of 7, and so after divvying out 21 whole pieces, the student represents giving out a piece of a piece by segmenting the final row of pieces to create shares of 3 and a little bit more for each friend. Alternatively, the student could have realized that 28 pieces was more pieces than were available to divvy up and crossed out the last row and given up. But in either case, based on these actions, we hypothesize that this student is not able to use composite units and anticipate the segmenting of the bar into 7 pieces but is aware that the bar will need to be broken into smaller pieces and shared equally. We further hypothesize that the similarity in length of the pieces was monitored by either visual estimates of similar size or by attempts to use a similar horizontal movement to get from each mark to the next.

In the next student example (Fig. 7b), a probable eTNS student appears to be attempting to coordinate the goal of making seven equal-sized pieces with the goal of exhausting the whole. We infer that the student tried the bottom attempt first, crossed it out, and then made the top attempt. We can see that the first (crossed-out) attempt to segment started with the right-hand segment, which was far too small, followed by the student’s attempt to equi-segment the remainder of the bar, keeping the segments about the same size. When the student sees that the resulting seventh piece is far too big, the student is then able to correctly determine that the first six pieces need to be longer and the last one shorter. The poor initial first guess at the necessary length for the segments indicates that the student is constrained to create the composite unit of 7 in activity before strategizing. The second attempt is similar and also, from the observer’s perspective, unsuccessful. We hypothesize that the student was aware of the great disparity that remained between the first and last segments and therefore aware that he or she did not meet the goal of making equal-sized pieces but decided to move on to the next task. This difficulty in planning accurate partitioning behavior observed in both attempts may be due to the fact that the probable eTNS student cannot use a mental re-presentation of seven to guide them in planning out the segmenting activity. When utilizing a mental re-presentation (e.g., Glasersfeld, 1991), students are able to mentally visualize seven partitions and then project them onto the given length of bar in order to make an estimate for where a partitioning line should be placed. While seven is a large number to clearly visualize, the simultaneous partitioner would be able to make an estimated visualization of seven partitions (or equally spaced objects) and “spread them out” until they exhausted the whole of the bar. Steffe refers to this use of re-presentation as a partitioning template (Steffe & Olive, 2010).

None of the probable INS or eTNS students made more than two attempts to partition the bar. While not all students categorized as INS or eTNS in our study have solutions like those shown in Fig. 7 (a and b), all but one of these students was unsuccessful in coming up with a good partitioning, which we judged by using a minimum and maximum size for each of the partitions.

Recall that a student who is using equi-partitioning understands the whole as resulting from iterations of a partitioned unit, a multiplicative relationship in which the whole is seen as *n* times as long as the partition. In contrast, a student who is using simultaneous partitioning understands that the partitions need to be equal in size, but the whole is not seen as resulting from iterations of one of the created units. The simplest evidence of the assimilatory use of composite units is the ability to partition the bar fairly well on the first (written) try. Note that the students whose solutions are shown in Fig. 7c–f make better initial estimates and better adjustments than the probable eTNS student. This pattern held for many probable aTNS and ENS students. Figure 7c, d shows such first attempts by probable aTNS students. Note these students also used their ability to simultaneously attend to all partitions to make efficient adjustments to their initial partitioning. In these solutions, students appear to make partitions all at once without estimation or prior to adjustment. In other words, each partition is made to be visually similar, instead of finding a unit that will exhaust the whole when iterated. This is evidenced by the lines that are marked out. These lines likely represent an initial attempt to divide the bar into 7 similar-sized pieces all at once. Once the student finishes, they recognize that the pieces are not visually similar and then adjust them to be smaller or larger to better represent equal-sized pieces. Figure 7e, f shows solutions by probable ENS students who evidence equi-partitioning through dots or small tick marks made to keep track of iterations of an initial partition in order to ascertain whether seven iterations produced the bar. We infer that this iterating behavior occurred before the final partitioning was drawn and that these dots would not be enough to help students visualize all seven pieces. This is a key difference between aTNS and ENS solutions that has been noted in teaching experiments and clinical interviews: Both groups of students can make good adjustments, but in the case of aTNS students, their goal is to make the seven pieces visually similar in size (Ulrich, 2016a, b; Steffe, 2010b). Therefore, they prefer to draw out all seven partitions and visually compare them to make their adjustments. ENS students who are equi-partitioning have been theorized (Steffe, 2010b) to have the goal of making sure the bar consists of seven iterations of the first partition, so they use fingers, dots, or tick marks to keep track of their iterations. They are not focused on visually comparing partitions.

Some aTNS and ENS students did not have a well-developed partitioning scheme, despite the fact that it would theoretically be within their zone of proximal construction to have constructed one. This was not surprising to us based on Lamon’s (1996, 2007) calls to include more partitioning opportunities in US classrooms beyond introductory activities in around third grade. As Watanabe (2007) points out, even the introductory activities often involve pre-partitioned figures, circumventing the need for partitioning behavior. The lack of experience many US students get with partitioning partially explains why it took many probable aTNS and ENS students multiple tries to get a good partitioning or why they did not attempt to make an accurate partitioning. In fact, the vast majority of probable aTNS and ENS students simply made a single partitioning and did not attempt to adjust it. If the original partitioning was not particularly accurate, we cannot glean much information about the students’ use of composite units in their partitioning schemes from these solutions.

#### Operating on composites of discrete units

Tasks involving discrete units in which figurative representations of units and (to the observer) composite units were provided seemed to elicit a greater variety of written solutions. We will use student solutions from two of these tasks to illustrate how use of composite units can be evidenced with written work. The first task was useful for differentiating the use of composite units in activity or assimilation. For this task, there are marked differences between the types of figurative support used by the students at different stages. The second task was useful for differentiating the use of composites of composite units in activity or assimilation.

**Fig. 8:**
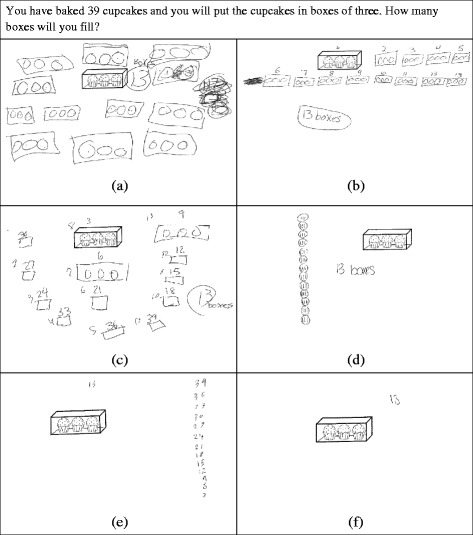
Student solutions to Cupcake Task A

Cupcake Task A (Figs. 1 and 6) was meant to be potentially accessible to all students in that students could draw out all 39 cupcakes and create triplets from there. In fact, two out of the four probable INS students drew out all the cupcakes, and three out of the four were able to successfully arrive at the answer of 13. Figure 8a is representative of both INS responses in which all cupcakes were drawn. In both, the student drew boxes of three cupcakes until 39 cupcakes were completed. In Fig. 8a, if you look closely, you can see that the student counted the individual cupcakes in the boxes, evidenced by pen marks in the circles. For the boxes that were drawn earlier, we can see numerous pen marks in the cupcakes, showing that the student counted those boxes numerous times. This indicates that the boxes did not stand in for composite units of three. Instead, the individual cupcakes were the predominant unit for the student. We hypothesize that the student did not attend to the boxes as units until all 39 cupcakes were counted. At that point, the student switched to counting boxes by 1. There is no indication that the student was aware of counting the number of 3s in 39. You can see that the drawing both here and in Fig. 8b (a probable eTNS solution) is very literal, with rectangles representing boxes and circles representing cupcakes.

Figure 8b is a solution from a probable eTNS student. It is similar to Fig. 8a, but the student does not give indications of recounting individual cupcakes. This implies that the student was keeping track of the 39 units of 1 as he or she made the boxes of 3 cupcakes: a simultaneous awareness of the composites of 3 and units of 1. Finally, the student numbers the boxes, which explicitly identifies them as countable units. Taken together, this gives strong indications that the student was constructing composite units of 3 in activity. Figure 8c shows a probable aTNS student who begins very similarly to the solution in Fig. 8b but who is able to curtail the production of individual cupcakes and only keep track of groups of 3. This points to an increasingly assimilatory use of composite units, consistent with advanced use of a TNS.

Figure 8d shows the solution of a probable aTNS student who uses a much less literal representation of the situation, with tally marks representing a counted cupcake and circles representing a completed box. Furthermore, this student seems to have been aware that he or she would be making multiple groups of 3 and counting them before drawing. This can be seen in the more organized nature of the drawing. Our hypothesis would be that this student was using the figurative material to keep track of the number of times three counts were made. This all implies an awareness of composite units of 3 in planning solution activity. Figure 8e shows the solution of a probable aTNS student who does not draw any figural representation of individual cupcakes. As in Fig. 8b–d, the student is working with composite units of 3, but the absence of units of 1 in the written work implies that these composite units did not need to be built up in activity, as in Fig. 8b–c. Note that aTNS students may use more literal representations. However, Fig. 8d, e shows that aTNS students are not constrained to using more literal representations.

Finally, some students (7 out of 44 students categorized as ENS; 2 out of 39 students categorized as aTNS) simply write the answer as in Fig. 8f. This implies the ability to assimilate the situation in terms of composite units of 3 and keep track of the number of composite units of 3 in the sequence from 1 to 39 with no figurative support. Clearly, this problem was relatively easy for aTNS and ENS students, so we will examine student solutions to a more complicated task that was meant to help differentiate between the TNS and ENS students.

**Fig. 9:**
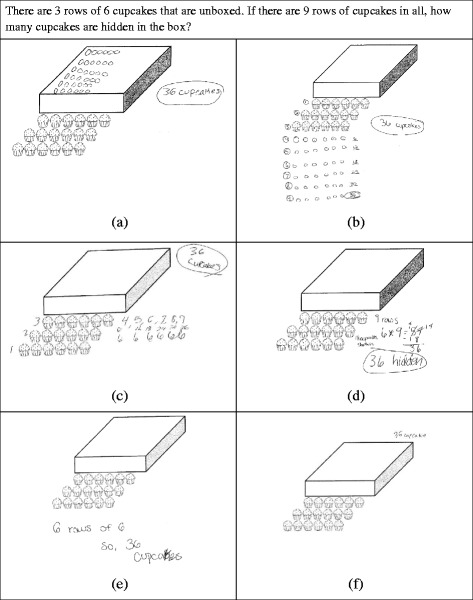
Six solutions to Cupcake Task C

In Cupcake Task C (Fig. 9), hidden cupcakes, shown cupcakes, and the total numbers of cupcakes are each composite units containing composite units (rows) of 6 cupcakes each. This complexity is meant to engender operations on composites of composite units. The quantitative complexity of the situation appears to be overwhelming for most INS or eTNS students, who usually leave the question blank or make a guess of how to combine the printed numbers: 3, 6, and 9. Only one eTNS (or INS) student successfully solved it after drawing all of the cupcakes in, similarly to the solution in Fig. 9a. The solutions shown in Fig. 9 are drawn from students categorized as aTNS (Fig. 9a–d) and ENS (Fig. 9e–f).

In Fig. 9a–d, we can see a common characteristic of aTNS solutions to these types of tasks: The students have all reduced the cognitive load of the problem by writing down intermediate calculations or using figurative material to keep track of hidden rows and/or cupcakes. In fact, in Fig. 9a, b, both students have drawn out all 36 hidden cupcakes. However, note that even in Fig. 9a, there is evidence of the use of a composite unit in assimilation, not just the experiential figurative composite formed by rows. In particular, the first two things the student seems to have drawn are (a) the first column of circles to represent the 6 hidden rows and (b) the entire top row. While the order of these actions is unclear, it is clear that the students operated on the composite unit of rows very early in solution activity in order to determine how many hidden rows there were before unpacking rows into their constituent units by drawing out the rest of the cupcakes in each row. Figure 9b more explicitly represents quantification of several units including the number of rows, the running subtotal of cupcakes in rows, and the total of shown cupcakes. Figure 9c, d was drawn by students who do not need the figurative support but do need to write down several intermediate calculations. In particular, in Fig. 9c, the student can now represent the composite unit of 6 cupcakes in a row by simply writing “6.” The composite unit does not need to be unpacked to show individual cupcakes. In Fig. 9d, the student also does not use figurative units of 1. However, note that the solution strategy simplifies the structure of the problem by immediately calculating the number of individual cupcakes in all so that the answer of 36 can be reached without attending to the number of hidden rows. In other words, for the student whose solution is shown in Fig. 9d, the composite unit of hidden cupcakes does not necessarily contain the intermediate composite units of 6 rows.

Another interesting aspect of the solutions in Fig. 9b, c is that the students show no indication of disembedding the 6 hidden rows and operating on that composite unit of rows separately. In fact, the 6 hidden rows are explicitly identified as the fourth through ninth row in both solutions. This is consistent with the general lack of reversible disembedding when working with a tacitly nested number sequence. In contrast, the solution by a probable ENS student in Fig. 9e, involves six hidden rows that are potentially disembedded from the sequence of nine total rows and operates on these six rows multiplicatively to determine the answer. Finally, Fig. 9f shows a common response for probable ENS students, who often only write the answer. In contrast, only one of the 39 probable aTNS students correctly answered this task without using an intermediate step or drawing. We claim that this is not simply indicative of a desire of the probable aTNS students to be thorough but rather is indicative of their need to decrease the cognitive load of the problem. This is further supported by the fact that even if aTNS students reduced the cognitive load, they were still more likely than students working at any of the other stage classifications to conflate quantities on this task. Presumably, this is because they have trouble keeping track of all of the levels of units involved in the problem but still attempt to do so. Note that ENS students might show work or drawings in order to be more thorough. Therefore, showing work is not a contraindication of an ENS, even though not showing work is an indication.

Cupcake Task C is indicative of six tasks on the assessment in which the student is given a situation that involves a composite of composite units. Generally, ENS students can operate effectively in these situations. Because of the lack of efficiency with which eTNS students conceptualize composite units, creating and then operating on composites of composite units creates an unsustainable cognitive load. However, aTNS students’ ability to anticipate the use of composite units opens the door for them to make sense of these tasks, even if they have trouble carrying out the mental operations to complete them effectively. This was evident in the fact that over 56% of students categorized as aTNS made a reasonable attempt at solving the majority of these tasks.

### Comparison of performance on the assessment and the bar tasks

Here we discuss the findings from comparing student performance on the bar tasks with stage classifications based on the rest of the assessment instrument (see Table 2). The data supported the hierarchy of bar task difficulty we had hypothesized; the overall percentages correct were as follows: B1, 82%; B2, 72%; B3, 71%; B4, 55%; and B5, 46%. Furthermore, the magnitude of the association between each of the tasks and the four stages were found to be positive, strong, and statistically significant based on the *G* statistic: B1, .76; B2, .72; B3, .62; B4, .77; and B5, .82. These associations are also reflected in the step-like patterns highlighted through shading in Table 2. These patterns highlight the stage classification for which tasks tend to become accessible. For example, the step-like pattern for task B2 suggests that the task is not accessible to INS or eTNS students but that the task becomes accessible to students operating with an aTNS.

**Table 2:**
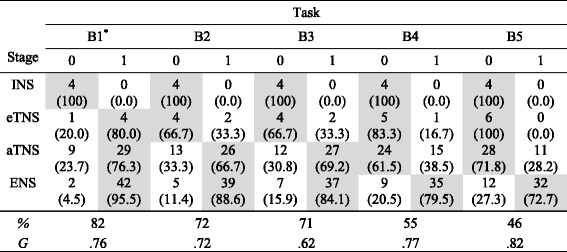
Association between performance on bar tasks and stage

*Note*: The numbers in parentheses represent the percentage of children answering correctly (1) versus incorrectly (0) for each task within stage.

*Two of the 93 students did not complete Task B1

However, further investigation of the contingency tables (see Table 2) revealed some unsuspected relationships: B1 (iterating a length of 3 units) corresponded in difficulty to the tasks that identify eTNS, not INS (as predicted by H3) and B2 (iterating a length of 8 units) corresponded in difficulty to tasks used to identify aTNS, not eTNS (as predicted by H4). When we created our hypotheses, we thought that INS students would be able to construct a numerical composite (supported by a visuospatial pattern, for example) for a small number, such as 3, in activity and then iterate that numerical composite in activity in order to solve B1. Similarly, we thought that eTNS students would be able to construct a composite unit in activity and then iterate it in order to solve B2. We furthermore thought that the presence of the numerical symbol for the composite—3 in the case of B1 and 8 in the case of B2—would be sufficient to elicit these constructions. However, it appears that an anticipatory use of composite units is necessary to recognize a single partition as representing a composite. Students did not appear to take the numeral as an impetus to construct a composite within the partition. In fact, this fits better with theory than our original hypotheses: Theoretically, an aTNS student is more advanced than an eTNS student precisely because they have an awareness of the results of producing composite units before carrying out the physical or mental actions that produce them. Without this awareness, there was no reason for INS or eTNS students to form the goal of constructing them to solve the bar tasks. Note that we are assuming students do not have difficulty in counting by 3s or carrying out repeated addition with 3 by this point in their mathematical career.

Another surprising result was that the presence of visible partitions in B2 did not greatly decrease the difficulty when compared to B3 (72 versus 71%). When iterating a composite unit, as in B2 and B3, there was only a net total of one more student who could solve the tasks when partitions (representing the number of iterations needed) were present. We had hypothesized that the need to determine the number of iterations needed would cause students who were creating composite units in activity (eTNS students) to conflate the various composite units at play. The fact that eTNS students were not able to solve either task would explain why B3 did not greatly increase in difficulty. When solving the reverse version of the bar task in which students were given the total length of the longer bar and asked to find the length of the shorter bar (the iterated composite unit), as in B4 and B5, the presence of partitions gave a jump in the number of correct attempts, a net total of 5 more students—1 more in eTNS and 4 more in aTNS—who correctly solved the task (consistent with H6, in that B5 was hypothesized to be substantially more difficult than B4, particularly for aTNS students). In this case, students who were coordinating composite units in activity included both aTNS and ENS students, and so the added level of complexity helped to distinguish those with more efficient unit structures (ENS students) from those who had less efficient unit structures and so were more likely to conflate the various types of composites.

Nonetheless, by far, the task difference that showed the greatest increase in difficulty was the transition from tasks in which students were given the size of the bar being iterated (B1–3) and those in which the size of the result of iterations was given (B4–5). While this does not contradict H5 because 38.5% of probable aTNS students were still able to solve B4, we were interested to see that a much higher percentage of ENS students (79.5%) solved it. Regardless, the jump in difficulty was not surprising in that students had to construct or assimilate with a composite unit made up of iterations of a composite unit and then further operate on that composite unit to solve B4. In the first three tasks, the student would not need to unitize the result of iterating because no further operation was necessary. Given the realizations discussed above and adjustment of our hypotheses, we found that the classified stages do align relatively well with the performance on the bar tasks. These relationships provide further evidence for the validity of the stage classifications.

## Conclusions

### Summary of findings

In our analysis of students’ written work on the assessment, we found that written work on tasks that included figurative unit items, such as cupcakes, boxes, and/or rows on the cupcake tasks, provided the greatest variety of evidence regarding students’ construction of and operation on composite units. Furthermore, a situation that would represent a simple division situation (Cupcake Task A) was most useful in helping determine whether the students had constructed composite units and/or were using composite units anticipatorily in planning solution activities. These distinctions are important for distinguishing between INS, eTNS, and aTNS students.

The other two cupcake tasks that involved reasoning about composites of composite units (groups of rows of cupcakes) were not generally solvable for INS or eTNS students, so they were in that way useful for differentiating between eTNS and aTNS. However, we focused on how correct solutions differed, which helped us distinguish between aTNS and ENS students. Overall, we found the following types of evidence in correct solutions of cupcake tasks: Solutions that lacked evidence of the construction of composite units (possible for INS students) included pen marks from counting and recounting units of 1, indicating a focus on counting units of 1 and not the figurative composites. Solutions that showed evidence of constructing composite units in activity (possible for eTNS students) included written counts of composite units, indicating the explicit awareness of composites as countable units. Solutions that showed evidence of an anticipatory use of composite units (possible for aTNS students) included (a) the complete or partial curtailment of drawing figurative units of 1 and/or a single symbol to represent a composite unit; (b) using composite units to organize diagrams (Figs. 8d and 9a); (c) written coordinations of multiple counts, such as counts of composite units and total units of 1; and (d) immediate unpacking of composites of composite units into units of 1, bypassing further operations on composite units. Solutions that indicated the ability to construct and potentially operate on in activity, composites of composite units (possible for ENS students) included (a) disembedding (lack of embedding) of a composite of composite units (ENS) and (b) the absence of written supports (no intermediate calculations or figurative items). It is important to remember that students may not use the most sophisticated strategies available to them on any particular problem, so that rubrics need to give more weight to the most sophisticated use of composite units that is evidenced. Moreover, because students’ written responses can only be interpreted as providing positive evidence or absence of evidence, the number sequence classifications for students based on these tasks represent a lower bound in terms of their reasoning with composite units.

We also examined written work on partitioning tasks to find indications of equi-segmenting (no indication of the use of composite units), simultaneous partitioning, or equi-partitioning. We found two types of solutions that showed equi-segmenting fairly clearly. In the first type of written work, students focused on breaking the bar into equal-sized pieces but did not attempt to match the number of pieces to the number of partitions desired. In the second type of written work, students made a poor estimate for the size of the first partition and then used that to make visually similar pieces. The final piece was often far too big or the correct number of pieces did not fit in the bar. This could be followed by an appropriate adjustment. However, we found that the students who gave clear indications of equi-segmenting and were probable INS or eTNS students did not do more than two attempts at segmenting and did not arrive at an adequate partitioning. We hypothesize that such students abandoned time-consuming equi-segmenting behavior because they lacked the ability to operate on a simultaneous awareness of all partitions in order to accurately adjust their approximations. Indications of equi-partitioning included curtailment of drawing partitions in favor of merely marking lengths to test out the appropriateness of a first partition’s length. In contrast, simultaneous partitioners drew out the partition and then adjusted the drawn partitions to even out their lengths. In many cases, it was difficult to determine the partitioning operations used on any one particular problem because students only made one partitioning with no intermediate attempts or marks evident. Therefore, written evidence is not as promising on these tasks.

We found that the hierarchy of difficulty for the bar tasks supported our conceptual framework. The tasks were useful in distinguishing between students at each of the four classifications. In particular, tasks in which an unpartitioned bar was identified with a larger composite unit were only successfully assimilated by probable aTNS students, implying that these tasks do a good job of testing a student’s ability to assimilate with composite units. Furthermore, we found that partitions that would scaffold a multiplicative comparison of the two bars did not greatly reduce the difficulty of the problems, implying that students who can assimilate a task with composite units do not have difficulty making and interpreting (what is to the observer) a multiplicative comparison of the composite units. Our findings imply that the bar tasks could be a powerful addition to assessments measuring a student’s stage in constructing and coordinating units.

### Implications

#### Future assessments

We believe that the findings in this study can help us and others better establish children’s current stage of ability to construct and coordinate units based on written work. One broad implication of our findings is that problems that include figurative unit items in the prompt could be particularly valuable in assessing student work. This could give practitioners, for example, additional ways to interpret student mathematical behavior outside of face-to-face questioning. It would also help communicate to practitioners what students at various stage classifications might “look like” in class. For example, if teachers know that students are consistently drawing out units of one, they may suspect that the student is working with an INS and can make appropriate interventions. Alternatively, a teacher may note that a student is able to curtail drawing out individual items, indicating the construction of composite units and a TNS.

Our more detailed findings about which problems students were able to independently solve, given their approximate stage classifications, and which problems seemed useful to differentiating between students at different stages, gives us and other researchers some insight into what types of problems may be useful when making a similar written assessment. Certainly, we have already used these results to figure out which items to keep and how to interpret student solutions in the rubric for the second iteration of this instrument.

#### Supporting the transition to multiplicative thinking

The transition from purely additive reasoning to multiplicative thinking and, ultimately, proportional reasoning is a key transition students are expected to make in middle-grade mathematics (see Lamon, 2007, 2012, for thorough discussions of these transitions). Just as proportional reasoning refers to more than the solving of proportions (Lamon, 2007, 2012), we use the term *multiplicative thinking* to refer to more than solving a multiplication problem. We refer to it as an awareness of a multiplicative relationship between two quantities.

As documented by Steffe (1992), even INS students can solve, what is to the observer, a multiplication problem. Certainly, TNS students can solve multiplication problems, and this corresponds to what Hackenberg and colleagues (e.g., Hackenberg & Lee, 2015; Hackenberg & Tillema, 2009) have popularized as the first multiplicative concept (see also Steffe, 1992). However, as Steffe (1992) explains, students with the first multiplicative concept cannot reinterpret the situation in terms of the results of their multiplicative unit coordination. That is to say, a student may determine the numerosity of “eight times seven” by counting by seven eight times and yet not be aware of the result, 56, as being in a multiplicative relationship with 7. The awareness of a multiplicative relationship does not start to emerge until the iterable unit of 1 that characterizes an ENS is constructed (e.g., Hackenberg & Lee, 2015; Ulrich, 2016a), and it is restricted to a multiplicative relationship between 1 and composite units at the ENS stage. Because this awareness of a multiplicative relationship is required for the type of multiplicative thinking outlined by Lamon (2012) in which students must be aware of not just the relationship but the intensive quantity representing that relationship (eight times as big as in this example), TNS students’ lack of awareness of multiplicative relationships bodes poorly for their ability to make a smooth transition to multiplicative thinking and proportional reasoning.

Therefore, we find students at the aTNS stage during middle school a particularly interesting and important group to identify and study because they have multiplicative schemes and yet will face a serious obstacle in trying to engage in truly multiplicative thinking. If we and other researchers could help teachers identify written indicators of this transition, that would give teachers one more way to be able to identify interventions for these students before they stumble in understanding proportional reasoning and algebraic symbolization of multiplicative relationships. Because aTNS students, in the cupcakes tasks, for example, can often get to the correct numerical answer and ENS students may give an incorrect numerical answer due to misreading, being attuned to other signals, like the amount of written work students use, can help teachers determine which students are which: aTNS students who may struggle with multiplicative thinking or ENS students who may be ready for such thinking. The large number of students classified as aTNS surprised even us. Given that aTNS students do not reason multiplicatively, this could have implications for appropriate teaching interventions at the sixth grade, where proportional reasoning is often introduced in the USA. However, we will need to further validate our instrument before making stronger claims.

### Limitations and future research

The findings from this study will be used to further refine our instrument and further validate its performance as an assessment tool. We have already used the results of this study to guide revisions of our assessment instrument and are currently revising our coding guide and scoring rubrics. We recognize a need for additional items that specifically focus on the differentiation between aTNS and ENS. In addition, we recognize a need to consider additional items that are more streamlined and less affected by extraneous factors unrelated to the assessment focus of the tasks. For example, in a future study, it might be helpful to test whether students’ facility with whole-number factor-product combinations is related to their performance when reasoning with composite units. By using multiple sets of bar tasks varied with different number combinations, we could test for this association, which may provide additional information for the selection of tasks for future assessment instruments.

We are also carrying out an increased number of clinical interviews to strengthen the validation efforts for our revised assessment and to test our hypotheses about what written evidence reveals about student thinking. The structured clinical interviews used in the current study constrained the interviewer from asking additional probing questions that could have provided additional evidence to make strong inferences about the number sequences available to the children. In our ongoing research, we are conducting semi-structured clinical interviews that allow for more focused follow-up questioning. This way, we may be better able to make strong inferences about children’s number sequences based on the interviews and, thus, stronger validity claims based on the match between findings from interviews and children’s written work.

This study provides an initial baseline estimate for the percentage of children classified with the different number sequences. However, the generalizability of this estimate is limited by the fact that our sample is made up of only sixth graders from one school. Thus, we would like to continue to look at these percentages as we refine our instrument and work with larger groups of students. Furthermore, we would like to see developmental patterns of middle-school children over time to give us further insight into needed learning support and appropriate curricular goals.

## Abbreviations

ENS: Explicitly nested number sequence
G: Gamma statistic
GNS: Generalized number sequence
INS: Initial number sequence
TNS: Tacitly nested number sequence

## Footnotes

1 In particular, we are looking at *arithmetic* units (Steffe, 2010a; Ulrich, 2015), which are interiorized counting acts.

2 It should be noted that this is not at all trivial for younger students who have constructed a TNS.

3 Note that fractions do not represent fully multiplicative relationships from the student’s perspective until they have constructed an iterative fraction scheme (IFS, see Steffe, 2010b). For example, a student with a partitive fraction scheme would interpret “3/5” as something like “3 pieces out of 5, any one of which could be iterated 5 times to form the whole.” In contrast, a student who had constructed an iterative fraction scheme would interpret “3/5” as something like “3 times the amount that can be iterated 5 times to form the whole.” In the case of the IFS, the numerator and the denominator represent multiplicative relationships, while only the denominator represents a multiplicative relationship for a student with a partitive unit fraction scheme.

4 We do not include the actual formulas and codes in this report as we are continuing to revise them based on new data and intend to publish more explicit discussion of the formulas in future work. The interested reader is encouraged to contact the authors for additional information about the formulas and codes.
